# Recombinant Antibodies to the Ebola Virus Glycoprotein

**Published:** 2017

**Authors:** A. A. Panina, I. G. Dementieva, T. K. Aliev, V. A. Toporova, D. S. Balabashin, M. N. Bokov, L. P. Pozdnyakova, O. B. Shchemchukova, D. A. Dolgikh, P. G. Sveshnikov, M. P. Kirpichnikov

**Affiliations:** Shemyakin-Ovchinnikov Institute of Bioorganic Chemistry, Russian Academy of Sciences, Mikluho- Maclay Str. 16/10, Moscow, 117997, Russia; Russian Scientific Center for Molecular Diagnosis and Treatment, Simferopol Blvd. 8, Moscow, 117149 , Russia; Lomonosov Moscow State University, Department of Chemistry, Leninskie gory 1, bldg. 3, Moscow, 119991, Russia; Lomonosov Moscow State University, Faculty of Biology, Leninskie gory 1, bldg. 12, Moscow, 119991 , Russia

**Keywords:** Ebola hemorrhagic fever virus, therapeutic recombinant chimeric antibodies

## Abstract

Currently, there are no approved therapies for targeted prevention and treatment of Ebola hemorrhagic fever. In the present work, we describe the development of a eukaryotic expression system for the production of three full-length chimeric antibodies (IgG1-kappa isotypes) GPE118, GPE325, and GPE534 to the recombinant glycoprotein of the Ebola virus (EBOV GP), which is a key factor in the pathogenicity of the disease. The immunochemical properties of the obtained antibodies were studied by immunoblotting and indirect, direct, and competitive ELISA using the recombinant EBOV proteins rGPdTM, NP, and VP40. The authenticity of the antibodies and the absence of cross-specificity with respect to the structural proteins NP and VP40 of the Ebola virus were proved. The epitope specificity of the resulting recombinant antibodies was studied using commercial neutralizing antibodies against the viral glycoprotein. The recombinant antibodies GPE118, GPE325, and GPE534 were shown to recognize glycoprotein epitopes that coincide or overlap with the epitopes of three well-studied neutralizing anti-Ebola virus antibodies.

## INTRODUCTION

The number of people that got infected during the most recent Ebola hemorrhagic fever outbreak exceeded 28,000, and more than 11,000 deaths were reported. Although the epidemic was stopped, the World Health Organization deems a new outbreak possible. In this regard, there is the urgent task of developing effective agents for the prevention and therapy of the disease.

Several approaches to the therapy of Ebola hemorrhagic fever have been proposed over the past decade. Thus, intravenous injection of inhibitors of blood coagulation, such as recombinant human-activated protein C, increases the survival rate in patients by 18% [1]. Intravenously administered small interfering RNAs increase the survival rate from 66 to 100% depending on the number of injections made [2]. However, the therapy needs to be started promptly, within the first 30–60 min post-infection.

Therapeutic antibodies against the Ebola virus have been under development since the 1990s. Among the eight viral proteins, the major pathogenicity factor, glycoprotein (GP), is considered to be the key immunotherapy target. Unstable results have been obtained when using certain monoclonal antibodies (mAT) or antibodies collected from the blood of Ebola survivors [3]. Meanwhile, G.G. Olinger Jr. et al. demonstrated in primate experiments that, unlike nonspecific antiviral therapeuticals, passive immunization with antibodies injected 24 h post-infection has a therapeutic effect [4]. There are ongoing attempts to find sources of neutralizing antibodies in the blood of Ebola survivors to develop agents that can be administered as monotherapy [5]. However, it was revealed during the production and study of anti-GP mAbs that the ZMapp antibody cocktail, a combination of antibodies specific to different GP epitopes, exhibits the strongest protective effect and significantly reduces lethality in model animals [6]. Different combinations of three GP-specific chimeric antibodies were tested in guinea pigs and rhesus macaques. The animals (18 rhesus macaques) were infected with lethal doses of the virus and were treated with ZMapp (50 mg/kg body weight) on days 3, 4, and 5 post-infection. ZMapp was found to ensure survival of all 18 rhesus macaques, including those who had strongly marked signs of the disease. The antibodies within the ZMapp cocktail were expressed in tobacco leaves and used during the 2014 Ebola epidemic in West Africa [7]. Compared to the group of patients receiving palliative care only, the mortality rate in the group receiving additional therapy with the study drug decreased from 37% (13 out of 35) to 22% (8 out of 36). Administration of the study drug in a larger patient sample was limited, because the trial was conducted at the final stage of the epidemic, when the number of newly infected patients was rather small, making it difficult to recruit Ebola virus carriers.

We previously obtained murine mAbs against the recombinant glycoprotein of the Ebola hemorrhagic fever virus (Zaire strain) lacking the transmembrane domain (rGPdTM) and selected three mAbs exhibiting different epitope specificities: GPE118, GPE325, and GPE534. The nucleotide and amino acid sequences of the variable domains were identified. The framework and hypervariable regions of immunoglobulin heavy and light chains were identified [8, 9].

The aim of this study was to design full-length recombinant chimeric antibodies against EBOV GP based on murine mAbs and to investigate their immunochemical properties: to determine the authenticity, specificity, and immunoreactivity of full-length chimeric antibodies and to measure the dissociation constants and the epitope specificity.

## EXPERIMENTAL

We used: the recombinant EBOV protein rGPdTM (IBT Bioservices, USA); recombinant proteins NP and VP40 (Fitzgerald Industries International, USA); mAb against human Ig kappa chain 4G7 and mAb against human IgG gamma-1 chain region (Hytest, Turku, Finland); anti-EBOV GP mAbs h13F6, c13C6FR1, c6D8, KZ52 and 4F3 (IBT Bioservices, USA); ExtrAvidin− Peroxidase conjugate (E2886, Sigma-Aldrich, USA); TMB substrate (3,3’,5,5’-tetramethylbenzidine, BioTest Systems, Russia); biotinyl-N-hydroxy-succinimide (H1759, Sigma-Aldrich); nitrocellulose membrane (Membrane filters, cellulose nitrate, pore size 0.45 μm, S045A330R, Advantec MFS, Inc., USA); Vivaflow 200 membrane (Sartorius Stedim Biotech, Germany); 96- well plates with high binding capacity (Corning-Costar, the Netherlands); and Tween 20.

**Construction of the expression system for chimeric mAbs in mammalian cells**

The chimeric sequences of light (L) or heavy (H) chains of mAbs were constructed by successively attaching the constant domain of the human kappa light chain (for the L chain) or the constant domains C_H_1-C_H_3 of the human IgG1 heavy chain (for the H chain) to DNA encoding the variable domain of the antibody. DNA fragments encoding the 5’-untranslated region and carrying a Kozak sequence, sequences of native leader peptides ensuring immunoglobulin secretion in the culture medium, and the 3’-untranslated DNA fragment carrying a polyadenylation site were also attached to the sequences listed above.

DNA fragments carrying the sequence coding for the variable domain of the heavy chain were PCR-synthesized on the template of plasmids produced earlier [8] and encoding the variable domain of the heavy chain of the antibodies GPE118, GPE534, and GPE325. SacI and ApaI restrictase recognition sites were simultaneously inserted at the 3’-end. For light chains, the mAb fragments forming a single transcription unit were joined using splicing by overlap extension PCR (SOE-PCR); the 5’-untranslated region and the leader peptide of heavy chains were attached using the same procedure. The constant domains C_H_1-C_H_3 of the human IgG1 heavy chain and the 3’-untranslated region carrying the polyadenylation site were ligated at the Apa I site that is present at the 5’-end of the human C_H_1 domain and was inserted to the 3’-end of the variable domain by PCR. The dual-promoter (the hEF1- HTLV promoter for the heavy chain and the CMV promoter for the antibody light chain) expression vector was constructed according to the procedure described in [10].

**Generation of cell lines producing recombinant antibodies**

Full-length recombinant antibodies were produced in CHO DG44 cells. The cells were cultured in a CD DG44 medium (Invitrogen) supplemented with 8 mM *L*-glutamine and 0.18% Pluronic F-68 (Invitrogen). A cell suspension (30 ml, 3 × 10^5^ cells/ml) was placed in 125 ml Erlenmeyer flasks and cultured under constant stirring on an orbital shaker at a rate of 130 rpm in a Sanyo MCO-18AIC CO_2_ incubator (Sanyo, Japan) at 37°C, under 8% CO_2_ and maximum humidity. Transfection was performed 24 h after the initiation of cultivation using Freestyle MAX reagent (Invitrogen). 15 µl of Freestyle MAX and 18 µg of the plasmid carrying the light and heavy chains of the antibodies under the control of the CMV and EF-1 alpha promoters, respectively, and the DHFR selection marker were used for transfection. Cell culture selection was carried out 48 h post-transfection. The cell culture was seeded into a nucleotide-free CD OptiCHO medium (Invitrogen). The cells were seeded into 125 Erlenmeyer flasks with 30 ml of the OptiCHO medium supplemented with 8 mM *L*-glutamine and cultured under the conditions described above. The first stage of selection in this medium was considered completed when culture viability of at least 95% was attained, while the cell population doubling time was 24 h. At the next stage of the selection of cell lines, 10 nM methotrexate (MTX) was added to the same culture medium. After this selection stage, experimental samples of recombinant antibodies started to be generated.

After selection using MTX, the producer cell lines were cloned on a ClonePIX FL cell sorter (Genetix, UK) to increase specific production of recombinant antibodies and stabilize the producers. The cell suspension was seeded onto a CloneMedia-CHO complete semi-solid medium supplemented with 8 mM *L-*glutamine and 10 mg/ml FITC-labeled human IgG secondary antibodies (Genetix, UK). Clones were selected according to the recommendations provided by the cell sorter manufacturer and cultured in a XP Media medium (Genetix, UK) supplemented with 8 mM *L*-glutamine. After the cell count had increased in each clone, the cells were transferred to the CD OptiCHO medium (Invitrogen, USA) for further growth, cryoconservation, and analysis of expression and growth properties.

**Generation of recombinant antibodies**

The cultures were seeded into 125 Erlenmeyer flasks containing 30 ml of the OptiCHO medium supplemented with *L*-glutamine to a concentration of 8 mM. Cell culture was started at a concentration of 3 × 10^5^ cells/ml under the conditions described above. Cultivation was terminated when cell culture viability decreased to 50%. The supernatant (0.5 l) with the verified presence of the antibody, which was obtained after cultivation of the CHO cell line, was centrifuged at 4,000 rpm for 30 min and passed through a filter with a 0.45 µm pore size. The supernatant was concentrated by tangential ultrafiltration on a Vivaflow setup (Sartorius, Germany) with a molecular weight cut-off < 50 kDa. The antibodies were isolated from the concentrated supernatant by affine chromatography on columns packed with protein A– agarose (GE Healthcare, USA) according to the manufacturer’s recommendations.

The content of recombinant antibodies in eluates after affine chromatography was evaluated by indirect ELISA.

**Immunoblotting of recombinant antibodies with mAbs 4G7 and 2C11**

Electrophoretic separation of recombinant antibodies (6 µg per lane) was carried out in 12% PAAG under reducing and nonreducing conditions. The proteins were then subjected to electrophoretic transfer (electroblotting) from the gel onto a nitrocellulose membrane with a 0.45 µm pore size (Advantec MFS, Inc., USA). The membrane was blocked with a solution of 5% casein in PBS overnight at 4°C and washed three times with PBS-T (10 mM K_2_HPO_4_, pH 7.5, 0.145 M NaCl, 0.05% Tween 20). The membrane was then cut into strips and incubated in antibody solutions (mAb 4G7 or mAb 2C11, 10 µg/ml, PBS) at 37°C for 1 h. After repeated five-fold washing with a PBS-T buffer, incubation in the presence of HRP-conjugated goat anti-mouse IgG secondary antibodies at a 1 : 15,000 dilution at 37°C was carried out for 1 h. After repeated three-fold washing in PBS-T, the substrate (3,3-diaminobenzidine, 4-chloro-1-naphthol, H_2_O_2_) was added and the system was incubated for 4–10 min. The reaction was stopped by washing the strips with water.

**Immunoblotting of the recombinant EBOV rGPdTM with recombinant antibodies**

After electrophoretic separation of EBOV rGPdTM (12 µg) in 12% PAAG under reducing conditions, electroblotting of the proteins from the gel to the nitrocellulose membrane and blocking as described above, the transferred proteins were detected on the nitrocellulose membrane by indirect ELISA (immunoblotting). For this purpose, the membrane was cut into strips, which were placed in a 10 µg/ml solution of the recombinant antibodies GPE118, GPE325, and GPE534 and incubated at 37°C for 1 h. After the incubation, the strips were incubated with HRP-conjugated mAb 4G7 at a dilution of 1 : 25,000 at 37°C for 1 h. The final wash and the reaction development were carried out as described above.

**Indirect ELISA with the immobilized recombinant Ebola virus proteins rGPdTM, NP, and VP40**

The antigen (1 µg/ml, PBS) was sorbed in the wells of a 96-well plate with high binding capacity at 4°C overnight. The plates were washed five times with PBS and 0.05% Tween 20. A solution of the recombinant antibodies under study (PBS, 2% BSA) was then added by threefold serial dilutions starting from a concentration of 3 µg/ml, incubated at 37°C for 1 h, washed five times with PBS and 0.05% Tween 20, and then incubated with HRP-conjugated mAb 4G7 against the human Ig kappa chain at a 1 : 50,000 working dilution (PBS, 2% BSA) at 37°C for 1 h. To develop the reaction, the wells were washed five times with PBS and 0.05% Tween 20; 100 µl of the TMB substrate was added to each well; and the plate was incubated at room temperature on a shaker for 15 min. The reaction was stopped with 0.5 M H_2_SO_4_, and absorbance was measured at a wavelength of 450 nm.

**Determination of K_d_ for the complex of recombinant antibodies and EBOV GP**

At the first stage, recombinant antibodies at a constant concentration of 7 pM (1 ng/mL) were incubated with the EBOV GP antigen in a concentration range of 0.1–10 nM (10–1000 ng/ml) at room temperature for 2 h under constant stirring to achieve thermodynamic equilibrium in the three-component system: free antigen, free antibody, and the antigen–antibody complex. At the second stage, the concentration of free recombinant antibodies was measured by solid-phase ELISA with EBOV GP immobilized on the plate. At the final stage, *K*d was calculated from the Klotz equation using the values of the total antigen concentration and the concentration of free recombinant antibodies [11].

**Competitive ELISA for epitope mapping of recombinant chimeric antibodies**

Parental murine mAbs, control commercial mAbs, and the full-length chimeric antibodies under study were sorbed at a concentration of 5 μg/ml at 4°C overnight in PBS, pH 7.2 on 96-well plates with high binding capacity (100 μl per well). The plate was washed 5 times with PBS-T (200 μl per well). The control mAbs KZ52, h13F6, c13C6FR1, c6D8, and 4F3 were titrated starting from a concentration of 6 μg/ml by three-fold serial dilutions in PBS-T (0.01 M KH_2_PO_4_, 0.1 M NaCl, 0.2% BSA, and 0.1% Tween 20), 50 μl per well. The recombinant viral protein was biotinylated using a fivefold excess of (+)-biotin N-hydroxysuccinimide ester (H1759 Sigma). Biotinylated EBOV rGPdTM was added at a concentration of 800 ng/ml in PBS-T, 50 μl per well. The plate was incubated at 37°C for 1 h with stirring. The plate was washed five times with PBS-T, 200 μl per well. ExtrAvidine–peroxidase conjugate at a concentration of 500 ng/mL was added to each well at a volume of 100 μl and incubated at 37°C for 1 h. The plate was washed five times with PBS-T, 200 μl per well. TMB substrate (100 μl) was added to each well. The plate was incubated at room temperature for 15 min on a shaker. The reaction was stopped by adding 100 μl of 0.5 M H_2_SO_4_ per well. The absorbance was measured at a wavelength of 450 nm.

## RESULTS AND DISCUSSION

Recombinant antibodies were produced using the suspension-adapted dihydrofolate reductase-deficient Chinese hamster ovary cells CHO DG44. The expression system was preliminarily constructed, and cell lines producing recombinant antibodies were generated for this purpose. A dual-promoter system based on the commercial vector pOptiVEC-TOPO containing the cytomegalovirus promoter (CMV) and hEF1-HTLV hybrid promoter for the translation of cloned genes, as well as the gene conferring resistance to geneticin, was used as an expression system in eukaryotic cell lines [10]. The vector also carries the gene coding for dihydrofolate reductase (DHFR), whose expression is regulated by the CMV promoter through the independent internal ribosome entry site (IRES). In case of coexpression of the target antibody gene, DHFR can be used as a selective marker to generate a stable cell line. DHFR expression makes it possible to neutralize the effect of the potentially cytotoxic methotrexate (MTX), thus maintaining cell proliferation. The *DHFR *gene is amplified at elevated MTX concentrations, which, in turn, increases the antibody gene copy number. Under selective pressure of methotrexate, genomic amplification of the genes of heavy and light chains occurs simultaneously. Proper dual-promoter constructs carrying nucleotide sequences coding for the kappa light chain and the IgG1 heavy chain were produced for each antibody.

The resulting CHO cell lines were used to generate recombinant antibodies by suspension cultivation in a serum-free medium. The quality of the experimental samples of anti-Ebola recombinant antibodies was controlled electrophoretically and chromatographically.

**Fig. 1:**
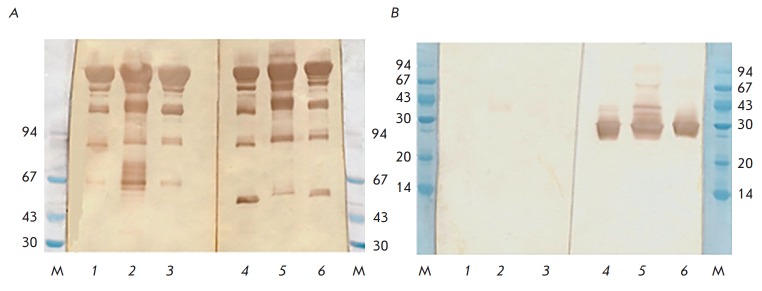
Immunoblot of recombinant chimeric antibodies under nonreducing (A) and reducing (B) conditions after 7% SDS-PAGE with conjugates based on mAbs 2C11 (lanes 1–3) and mAb 4G7 (lanes 4–6). Lanes 1, 4 – GPE 118; lanes 2, 5 – GPE 325; lanes 3, 6 – GPE 534. Lane M – molecular weight standards, kDa.

The authenticity of the antibodies was proved by immunoblotting using monoclonal antibodies specific to the characteristic regions of the heavy and light chains: murine mAbs 4G7 against human Ig kappa chain and mAbs 2C11 against human IgG gamma-1 chain. Immunoblotting was performed under nonreducing and reducing conditions. The results are shown in *Fig. 1*.

The immunoblotting data demonstrate that all the samples of recombinant chimeric antibodies under nonreducing conditions yield a major band with mobility corresponding to a molecular weight of ~150 kDa and become virtually identically stained with mAbs 4G7 and 2C11, which corresponds to the composition and the anticipated molecular weight of an IgG molecule. All antibody samples also give three or four additional minor bands that become stained with both mAbs and contain fragments of the heavy and light chains, being indicative of potential proteolytic degradation of antibodies, incomplete assembly (the absence of closing of disulfide bonds in the antibody hinge region), and presence of free light chains. Under reducing conditions, the antibody 4G7 stains only the kappa free light chain with electrophoretic mobility corresponding to ~25 kDa. Antibody 2C11 against the human IgG1 heavy chain produces no bands in antibody samples under re ducing conditions, which demonstrates that binding of this antibody depends on closure of intrachain disulfide bonds in the antibodies under study. Mobility and the band pattern revealed by immunoblotting using 2C11 and 4G7 antibodies unambiguously prove the authenticity of the samples of the full-length recombinant anti-EBOV GP antibodies GPE118, GPE325, and GPE534.

**Fig. 2:**
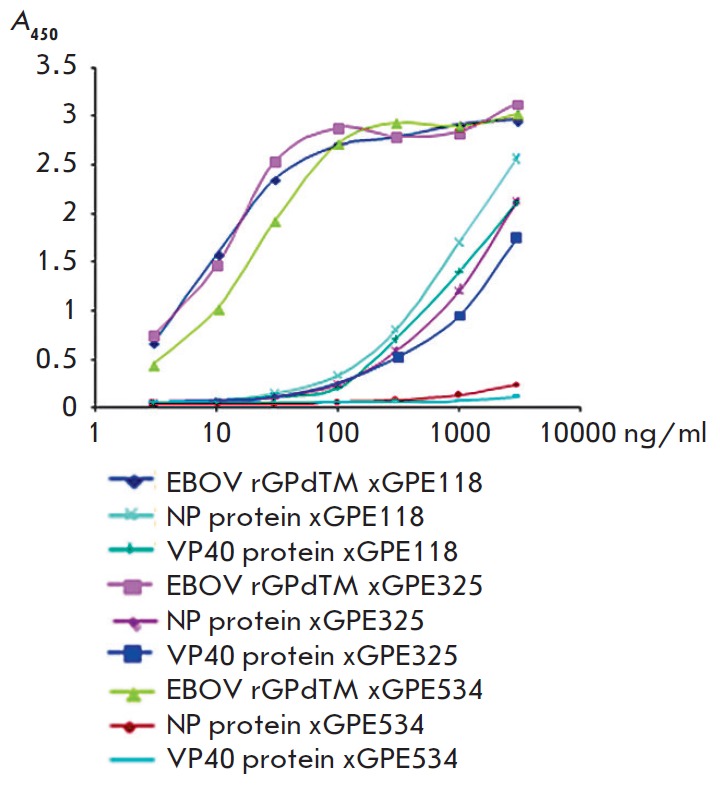
Titration curves of full-length chimeric antibodies in indirect ELISA (OD 450 nm) with the immobilized EBOV proteins rGPdTM, NP, and VP40. xGPE118, xGPE325, and xGPE 534 – chimeric recombinant antibodies against rGPdTM; NP – nucleoprotein; VP40 – structural protein; and rGPdTM – the Ebola virus glycoprotein.

The immunoreactivity of recombinant chimeric anti-EBOV GP IgGs was determined by indirect ELISA with the immobilized EBOV proteins rGPdTM, NP, and VP40 (*Fig. 2*).

The results of indirect ELISA of full-length chimeric antibodies with the immobilized structural EBOV proteins rGPdTM, NP, and VP40 demonstrate that all these antibodies are targeted against the viral glycoprotein only

**Fig. 3:**
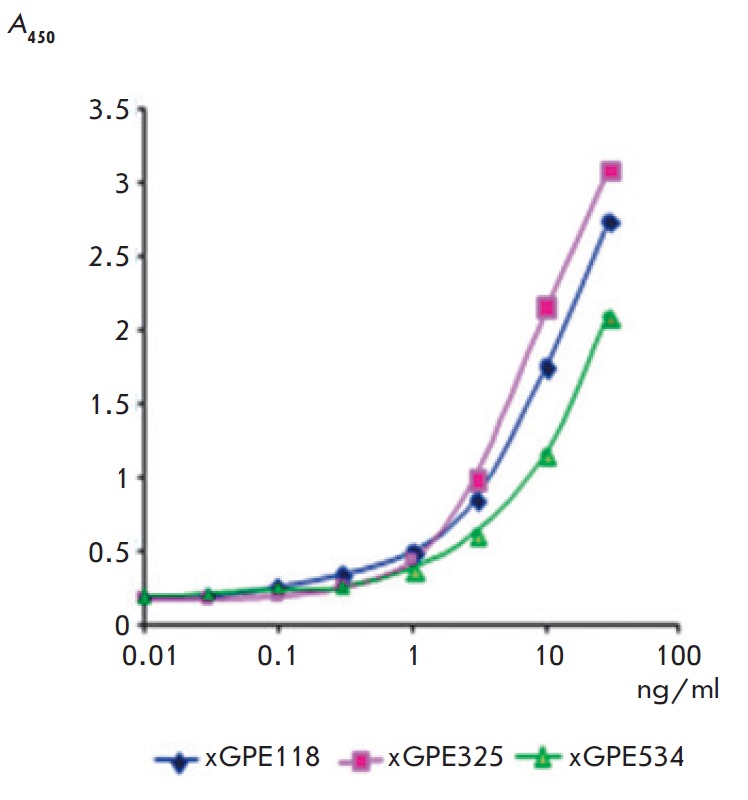
Absorbance at 450 nm as a function of the concentration of full-length chimeric antibodies in indirect ELISA with immobilized EBOV rGPdTM. xGPE118, xGPE325, xGPE 534 – chimeric recombinant antibodies against rGPdTM

The immunoreactivity (determining the end point titer, EPT) of full-length chimeric antibodies was evaluated by indirect ELISA with sorption of EBOV rGPdTM onto the solid phase. The results of titration (*Fig. 3*) of full-length chimeric antibodies in indirect ELISA with immobilized EBOV rGPdTM demonstrate that the experimental samples have a high affinity for the target protein: the EPT values for xGPE118, xGPE325, and xGPE534 correspond to a concentration of 0.3 ng/ml (2 pM).

**Fig. 4:**
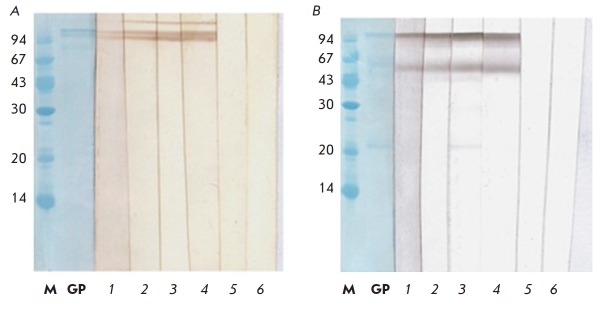
Immunoblot of recombinant chimeric antibodies against EBOV GP after 12% SDS-PAGE under nonreducing (A) and reducing (B) conditions. After transfer to the membrane, each strip was incubated with a separate antibody. Lane 1 – GPE 118, lane 2 – GPE 325, lane 3 – GPE 534, lane 4 – mAT c6D8 (positive control), lane 5 – h13F6 (negative control), lane 6 – FI6v3, lane M – molecular weight standards, kDa

The specificity of three experimental samples of full-length recombinant chimeric anti-EBOV GP antibodies was analyzed by immunoblotting (*Fig. 4*) using recombinant EBOV rGPdTM, which is the extracellular part of the viral glycoprotein with a deleted transmembrane domain (this domain was expressed in insect cells and has a natural glycosylation pattern).

The immunoblot results demonstrate that the full-length recombinant antibodies GPE118, GPE325, and GPE534 give a single major band with a molecular weight of ~95 kDa under nonreducing conditions, which corresponds to full-length glycoprotein GP. Under reducing conditions, these antibodies yield two bands corresponding to the GP1 and GP2 subunits, thus unambiguously proving the specificity of the resulting antibodies against EBOV rGPdTM.

The dissociation constants of the antigen–antibody complex were determined in Klotz coordinates using the method suggested by Friguet et al. [11]. *Table 1* compares the dissociation constants of parental murine mAbs [9] and the full-length recombinant chimeric proteins produced.

Comparison of the affinities of the parental murine mAbs and recombinant chimeric antibodies shows that this parameter remains unchanged as one proceeds from natural full-length mAbs to full-length recombinant chimeric antibodies, although the constant antibody domains have been modified, thus being indicative of proper identification of the amino acid sequences of murine mAbs and the proper folding of recombinant proteins in the selected expression system. We would like to mention that affinity of the IgG1 antibody xGPE325 was even somewhat higher than that of parental murine IgM mAbs.

Full-length recombinant antibodies were used for epitope mapping by competitive ELISA using commercial antibodies with the known epitope specificity (*Table 2*). This analysis is needed to theoretically assess the potential protective activity. The first step in verifying proper selection of three and more anti-EBOV GP monoclonal antibodies is to demonstrate that mAbs are bound to or interact with three nonoverlapping epitopes of GP and that these epitopes are close to those of the known neutralizing antibodies.

When conducting solid-phase competitive ELISA using a monomeric antigen of biotinylated EBOV rGPdTM, the capture antibody under study was immobilized on a solid phase carrier; the biotinylated antigen and control mAb with the known epitope specificity were added simultaneously. If each mAb within a pair is targeted against different (nonoverlapping) recognition sites (epitopes), the three-component complex capture antibody–antigen–control mAb is formed. No three-component complex is formed on the solid phase if both antibodies are targeted against the same epitope.

**Table 1:** Comparison of K_d_ for parental murine mAbs and full-length chimeric antibodies

Sample	Subisotopes	K_d_, nM
GPE118	IgG1 kappa, mouse	1.7–2.0
xGPE118	IgG1 kappa, human	2.5–4.0
GPE325	IgM kappa, mouse	1.8–3.4
xGPE325	IgG1 kappa, human	1.2–2.5
GPE534	IgG2b kappa, mouse	0.8–1.0
xGPE534	IgG1 kappa, human	1.3–1.9

Note. хGPE118, хGPE325, хGPE534 – chimeric recombinant antibodies against rGPdTM.

Each commercial antibody was immobilized on a solid-phase carrier. Competitive ELISA was performed using the recombinant chimeric antibodies GPE118, GPE325, and GPE534, as well as commercial antibodies with a known epitope specificity [12]. The results of competitive ELISA for the produced full-length recombinant chimeric and commercial antibodies upon binding to biotinylated EBOV rGPdTM allow one to qualitatively characterize the epitopes of the antibodies under study. The antibody GPE534 competes with the neutralizing antibody KZ52; the antibodies GPE118, GPE325, and GPE534 strongly, although to a different extent, compete with the neutralizing antibody h13F6. The antibodies GPE118 and GPE534 compete rather weakly with the neutralizing antibody c13C6 and weakly compete with the neutralizing antibody c6D8. None of the antibodies under study competes with non neutralizing murine mAb 4F3. The competitive ELISA data (not shown) allow one to calculate the coefficient of inhibition (CI) for the binding of full-length recombinant antibodies with biotinylated EBOV rGPdTM in the presence of control commercial antibodies at different concentrations (*Table 3*). CI is the ratio between absorbance in competitive ELISA in the presence (3 µg/ml) and in the absence of the control mAbs. At CI ≥ 1, there is no competition between the control mAb and the full-length antibodies under study; i.e., the antibodies are targeted against different epitopes. If CI values are below 1, the control mAb and the full-length antibodies under study interact with the same or the closely located epitopes. The smaller the CI value, the closer the epitopes are located.

**Table 2:** Properties of the commercial anti-EBOV GP antibodies used in this study

Antibody	Species	Epitope	Polypeptide	Component of the antibody cocktail	Neutralizing activity	Reference
KZ52	Human	conformational	GP1–GP2	none	+	[11]
h13F6	Mouse/human	404–412	GP1	MB-003	+	[4]
c13C6 FR1	Mouse/human	33–295	GP1	MB-003, ZMapp	+	[4, 5]
c6D8	Mouse/human	393–401	GP1	MB-003	+	[4]
4F3	Mouse	NA		none	-	

An analysis of the CI values allows one to infer that the epitopes of the full-length recombinant epitopes GPE118, GPE325, and GPE534, chimeric Fab fragments [13], and the parental murine mAbs coincide. All three candidate anti-EBOV GP antibodies are also targeted against different epitopes. None of them competes with nonneutralizing mAb 4F3. The epitope of the antibodies GPE118 and GPE325 overlaps with the epitope of mAb h13F6 localized in the EBOV glycoprotein mucin-like domain between the amino acid residues 404–412 of the GP1 glycoprotein subunit. GPE118 shows stronger competition for binding to the antigen than the antibody h13F6 competes with itself. The epitope of the antibody GPE325 overlaps with the epitope of mAb c6D8 localized between the amino acid residues 393–401 of the GP1 subunit and is slightly shifted towards the N-end of the GP1 subunit with respect to the epitope of GPE118. The epitope of GPE534, having a linear nature according to the immunoblotting data, resides near the conformational epitope of mAb KZ52 formed by numerous amino acid residues of the GP1 and GP2 subunits of the viral glycoprotein. Hence, all three full-length recombinant chimeric anti-EBOV GP antibodies have epitopes that either coincide or overlap with the epitopes of the three well-studied neutralizing anti-EBOV mAbs.

**Table 3:** The coefficient of inhibition of experimental samples of the full-length antibodies GPE 118, GPE 325, and GPE 534 by the control mAbs according to the data of competitive ELISA with biotinylated EBOV rGPdTM

Control mAbs	CI of the control mAb	GPE118 IgG/Fab*/mAb	GPE325 IgG/Fab*/mAb	GPE534 IgG/Fab*/mAb	Epitope, a.a.r.	Polypeptide
h13F6	0.39	0.35/0.69/0.31	0.41/0.57/0.30	0.96/1.00/0.97	404–412	GP1
c13C6FR1	0.20	0.86/0.93/0.94	1.13/0.94/1.18	0.96/1.01/0.88	33–295	GP1
c6D8	0.39	0.76/0.99/0.77	0.60/0.64/0.70	0.67/0.99/0.97	393–401	GP1
KZ52	0.17	1.09/1.02/1.03	1.04/1.10/1.11	0.63/0.92/0.86	Conf.	GP1-GP2
4F3	0.43	1.05/0.90/1.23	1.31/1.10/1.44	1.0/0.87/0.96	NA	NA

^*^According to the findings reported in [13].

Note. contr. mAbs – control mAbs; IgG – full-length chimeric antibodies; conf. – conformational; NA – no data available.

## CONCLUSIONS

Full-length recombinant chimeric antibodies against the Ebola virus glycoprotein GPE118, GPE325, and GPE534 were constructed and produced in CHO cells. The immunochemical properties of the full-length recombinant chimeric antibodies were studied by immunoblotting and indirect, direct, and competitive ELISA using the recombinant EBOV proteins rGPdTM, NP, and VP40. The authenticity of the full-length recombinant chimeric IgG1 antibodies and their specificity with respect to EBOV GP were proved by immunoblotting using mAbs 4G7 against the human Ig light chain kappa and mAbs 2C11 against the human IgG1 heavy chain. The results of indirect ELISA demonstrate that there is no cross-specificity with respect to the proteins NP and VP40 of the Ebola virus. The affinity of the full-length antibodies shows that the experimental samples exhibit high affinity to EBOV GP.

An analysis of the coefficients of inhibition determined by competitive ELISA using a panel of commercial neutralizing antibodies allows us to draw the conclusion that all the antibodies under study are targeted against different glycoprotein regions. The epitopes of the recombinant antibodies either coincide or partially overlap with the epitopes of three commercial neutralizing anti-Ebola virus antibodies. This finding demonstrates that the resulting recombinant antibodies exhibit a potentially high neutralizing activity.

## Abbreviations

mAbs: monoclonal antibodies
EBOV: Ebola hemorrhagic fever virus
EBOV GP: glycoprotein of the Ebola hemorrhagic fever virus
rGPdTM: recombinant glycoprotein of the Ebola hemorrhagic fever virus lacking transmembrane domain
NP: Ebola virus nucleoprotein
VP40: structural protein of the Ebola virus
SOE-PCR: splicing by overlap extension PCR
IEDB: the Immune Epitope Database
Kd: the dissociation constant
PBS: phosphate buffered saline
